# Workload of pharmacists and the performance of pharmacy services

**DOI:** 10.1371/journal.pone.0231482

**Published:** 2020-04-21

**Authors:** Shih-Chieh Shao, Yuk-Ying Chan, Swu-Jane Lin, Chung-Yi Li, Yea-Huei Kao Yang, Yi-Hua Chen, Hui-Yu Chen, Edward Chia-Cheng Lai

**Affiliations:** 1 Department of Pharmacy, Keelung Chang Gung Memorial Hospital, Keelung, Taiwan; 2 School of Pharmacy, Institute of Clinical Pharmacy and Pharmaceutical Sciences, College of Medicine, National Cheng Kung University, Tainan, Taiwan; 3 Department of Pharmaceutical Material Management, Chang Gung Medical Foundation, Taoyuan, Taiwan; 4 Department of Pharmacy Systems, Outcomes & Policy, College of Pharmacy, University of Illinois at Chicago, Chicago, Illinois, United States of America; 5 Department of Public Health, College of Medicine, National Cheng Kung University, Tainan, Taiwan; 6 Department of Pharmacy, Linkou Chang Gung Memorial Hospital, Taoyuan, Taiwan; 7 Department of Pharmacy, National Cheng Kung University Hospital, Tainan, Taiwan; College of Pharmacy & Health Sciences, UNITED STATES

## Abstract

**Objective:**

To evaluate the influence of pharmacists’ dispensing workload (PDW) on pharmacy services as measured by prescription suggestion rate (PSR) and dispensing error rate (DER).

**Method:**

This was an observational study in northern and southern Taiwan’s two largest medical centers, from 2012 to 2018. We calculated monthly PDW as number of prescriptions divided by number of pharmacist working days. We used monthly PSR and DER as outcome indicators for pharmacists’ review and dispensing services, respectively. We used Poisson regression model with generalized estimation equation methods to evaluate the influence of PDW on PSR and DER.

**Results:**

The monthly mean of 463,587 (SD 32,898) prescriptions yielded mean PDW, PSR and DER of 52 (SD 3) prescriptions per pharmacist working days, 30 (SD 7) and 8 (SD 2) per 10,000 prescriptions monthly, respectively. There was significant negative impact of PDW on PSR (adjusted rate ratio, aRR: 0.9786; 95%CI: 0.9744–0.9829) and DER (aRR: 0.9567; 95%CI: 0.9477–0.9658). Stratified analyses by time periods (2012–2015 and 2016–2018) revealed the impact of PDW on PSR to be similar in both periods; but with positive association between PDW and DER in the more recent one (aRR: 1.0086, 95%CI: 1.0003–1.0169).

**Conclusions:**

Reduced pharmacist workload was associated with re-allocation of pharmacy time to provide prescription suggestions and, more recently, decrease dispensing errors. Continuous efforts to maintain appropriate workload for pharmacists are recommended to ensure prescription quality.

## Introduction

Medication errors may cause unintended treatment outcomes or mortality[1,2]. There are two major types of medication errors, including dispensing errors and medication prescribing errors, accounting for 4% and 70% of medication errors, respectively[3,4]. Dispensing errors are defined as discrepancies between the medications that were prescribed and those that were dispensed. Medication prescribing errors are defined as errors from prescribing decisions or prescription writing processes resulting in unintentional reductions of treatment effects or possible harms to patients[4,5]. It is important to establish strategies for the reduction of medication errors to maintain high quality and safety of treatment[6,7].

Pharmacists play an indispensable role in delivering drugs and assuring their proper use[8–10] when medication is being used more widely. Over the past decades, the pharmacist profession has expanded its role from dispensing medication to providing consultations to patients, and taking clinical responsibility for ensuring successful patient care outcomes, such as by decreasing adverse drug events and medication errors[11,12]. However, the escalating use of prescription drugs nowadays has significantly increased the pharmacists’ workload and compelled them to spend more time on the traditional dispensing role. Although automation may increase dispensing efficiency, the overall demand for pharmaceutical care remains high[13]. A study found that more than two-thirds of pharmacists considered their workload to be excessive[14], and some studies indicated an association between a high workload and poor quality of pharmacy services, as indicated by the failure to detect prescribing errors with drug-drug interactions and dispensing errors[15–18]. However, there has been no prior study to evaluate the impact of pharmacy workload on both drug dispensing and prescription validation, which share the same clinical importance for pharmacists with regard to medication quality and safety.

Different from the medical systems of other countries, pharmacists in Taiwan are in charge of all dispensing procedures and assistance from pharmaceutical technicians is prohibited. Due to the accessibility of medicine supported by the Taiwan National Health Insurance Program, the number of prescriptions and the related workload for pharmacists may have greatly increased over time[13]. A previous survey in Taiwan reported that dispensing overload was the top burden for hospital pharmacists, and it was also associated with adverse work outcomes[19]. Government and hospital administrators in Taiwan have undertaken great effort to reduce the dispensing workload of pharmacists in pursuit of better pharmacy services. For example, hospital accreditations from the Taiwan Ministry of Health and Welfare stipulate an optimal dispensing volume of 40 inpatient prescriptions or 70 outpatient prescriptions per pharmacist per day for medical centers, and hospitals have introduced many health information technologies to assist pharmacists in reviewing and dispensing prescriptions.

There is much evidence on how the increased workload negatively impacts pharmacy services, but there has been no study to evaluate if reduced workload of pharmacists improves the quality of pharmacy services. In this study, we evaluated the impact of pharmacists’ dispensing workload (PDW) on pharmacy services using two indicators, prescription suggestion rate (PSR) and dispensing error rate (DER). Based on previous studies of negative impact of workload on pharmacy services, we hypothesize that an increase in PSR and a decrease in DER may result from efforts to reduce pharmacist workload.

## Methods

### Data source

This is a secondary analysis of observational data collected from the two largest medical centers in northern and southern Taiwan; together, they have more than 6,300 beds, and 200,000 hospitalizations and 5,500,000 outpatient visits each year[20,21]. To maintain the quality of prescriptions and drug safety, the hospitals in 1993 established an electronic reporting system for pharmacists to submit their recommendations to clinicians regarding the appropriateness of prescriptions and to record dispensing errors from ambulatory, emergency and inpatient settings throughout the system. In addition, starting in 2009, the study hospitals implemented electronic review of prescriptions with the assistance of computerized physician order entry with clinical supportive systems (CPOE-CSS), and in 2011 introduced more complete CPOE-CSS functions, such as the detection of serious drug-drug interactions and drug allergy. The intensification of CPOE-CSS for these detections, such as cross-institutional review of prescriptions, was performed during 2013–2015. To improve the reporting rate of prescription suggestions and dispensing errors, study hospitals have encouraged each report of prescription suggestion with a reward of up to 100 NT dollars (approximately 3.3 USD). In order to encourage pharmacists to report dispensing errors, hospitals instructed pharmacy directors to deduct points for unreported dispensing errors when evaluating pharmacists’ performance. All reports were validated by two independent pharmacists to ensure the completeness and accuracy of records, and the reporting systems were identical in these 2 study hospitals. This study was approved by the Institutional Review Board at Chang Gung Medical Foundation, Taoyuan, Taiwan (No. 201700310B0).

### Study design and outcome indicators

**Fig 1:**
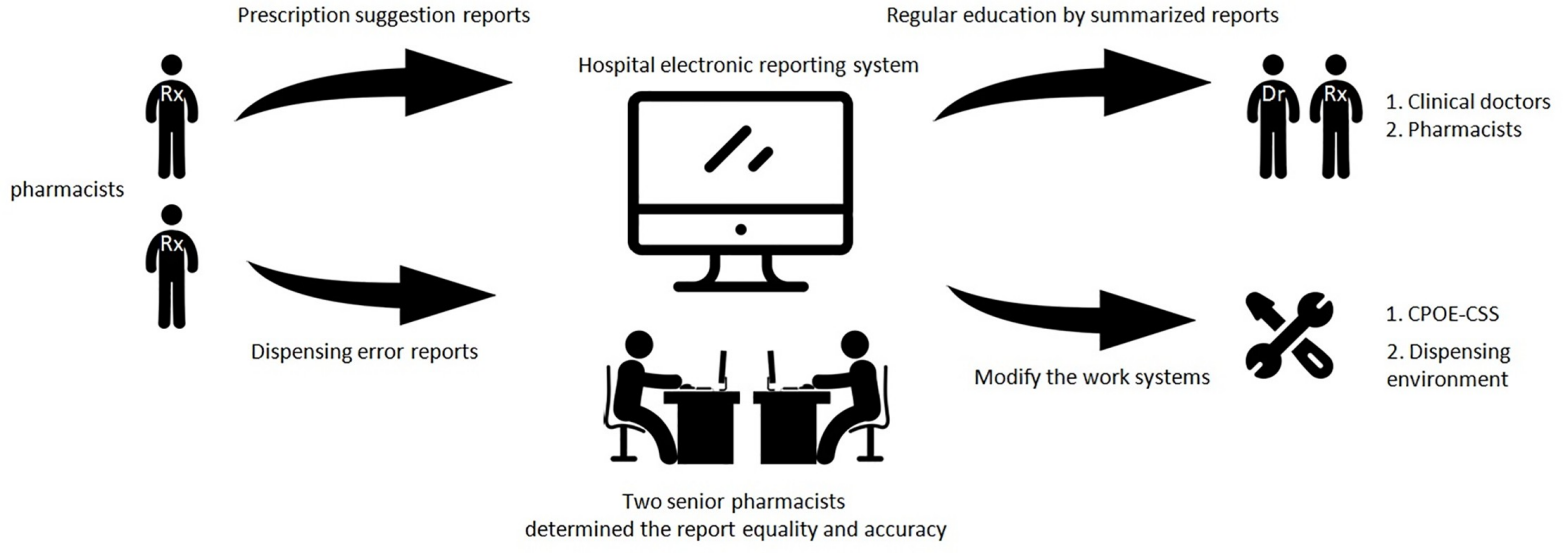
The process of pharmacists’ prescription suggestions and dispensing reports being sent to the systems and further quality assurances in study hospitals. CPOE-CSS, computerized physician order entry with clinical supportive systems.

We evaluated PDW by calculating the average number of prescriptions per pharmacist working days per month from 2012 to 2018. We measured the clinical services of pharmacists by two indicators on a monthly basis: 1) the PSR, which was the number of suggestions per 10,000 prescriptions and 2) the DER, which was the number of dispensing errors per 10,000 prescriptions. A high PSR is an indication that pharmacists can review prescriptions in detail to discover any potential drug-related problems, while a low DER indicates that pharmacists are able to provide the correct drugs to patients based on the prescriptions, both of which are major responsibilities of pharmacists in order to maintain drug quality and safety of patients. These two indicators were reviewed monthly by two senior pharmacists and used for internal quality assurance for pharmacist services in the study hospitals (**Fig 1**).

Moreover, we classified the pharmacists’ prescription suggestions into two groups depending on whether or not the suggestions were accepted by the prescribing doctors. Compared to prescription suggestions not accepted by prescribing doctors, it took more time for pharmacists to provide accepted prescription suggestions regarding drug-related problems because in most cases pharmacists needed to recommend alternative drugs or possible solutions to doctors. At the same time, we recognized an important value of the prescription suggestions not accepted by doctors to lie in the form of prescription review by pharmacists, double checking the quality of drug uses. Furthermore, we classified the accepted suggestions from pharmacists into three levels based on the time required for the review by pharmacists: 1) Prescription suggestions in response to entry errors in the CPOE-CSS. These errors were made by doctors while entering the prescriptions into the computerized system. This group of errors generated the simplest type of suggestions and consumed the least amount of time of a pharmacist; 2) Prescription suggestion alerts raised by the CPOE-CSS. The automatic alerts from CPOE-CSS included the detection or validation of drug allergy or drug-drug interaction. This group of errors would cost more time for a pharmacist to review the potential drug-related problems; 3) Prescription suggestions not alerted by the CPOE-CSS. These drug-related problems were those that could not be automatically detected by the CPOE-CSS, and the review of the appropriateness of these prescriptions would require the most time from a pharmacist. An example would be a pharmacist making a suggestion to adjust the dose of an antibiotic based on the renal function of a specific patient. We also assessed the influence of PDW on different types of dispensing errors, including 1) wrong drugs dispensed and 2) wrong drug amount dispensed.

### Statistical analyses

Descriptive statistics were used to summarize characteristics of dispensing workload and pharmacy services in the study hospitals. All variables were described by using the mean and standard deviation (SD). We assessed the association of PDW with PSR and DER using the Poisson regression model with generalized estimation equation method, which accounts for the inter-correlation of monthly data within a hospital.

The interaction of time and hospital was further included to assess whether the associations of PDW with PSR and DER varied with study hospitals. During the study periods, the study hospitals underwent hospital re-accreditations by the Taiwan Ministry of Health and Welfare, complying with the sixth-stage HIMSS model in 2016. To determine the effects of PDW on PSR and DER in different time periods, we also analyzed the stratified models for two different time periods (e.g., 2012–2015 and 2016–2018). All P-values were 2-sided and those less than 0.05 were considered statistically significant. We performed analyses using SAS Enterprise statistical software (version 5.1).

## Results

**Fig 2:**
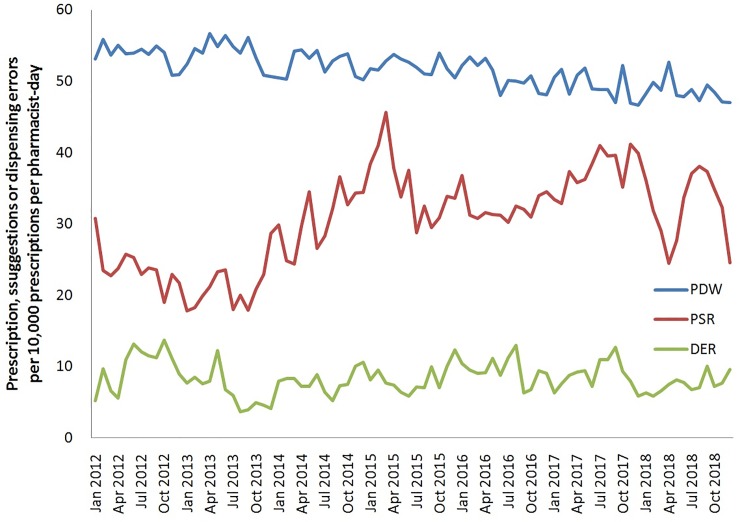
The trends of PDW, PSR and DER between 2012 and 2018. PDW, pharmacist dispensing workload; PSR, prescription suggestion rate; DER, dispensing error rate.

The monthly mean number of pharmacists included in the study was 394 (SD 17), who contributed to the mean number of 9,016 (SD 796) pharmacist working days from 2012 to 2018. With a monthly mean amount of 463,587 (SD 32,898) prescriptions dispensed, pharmacists provided a mean number of 1,410 (SD 324) prescription suggestions and made a mean number of 388 (SD 112) dispensing errors (**Table 1**). The mean PDW was 52 (SD 3) prescriptions per pharmacist working days, the mean PSR was 30 (SD 7) and DER was 8 (SD 2) per 10,000 prescriptions. We present the trends of PDW, PSR and DER in hospitals during the study period in **Fig 2 and S1 Table**, whereby there appeared to be similar trends within the individual hospitals (**S1 Fig**).

**Table 1:** Pharmacists’ workload and pharmacy services in study hospitals during 2012–2018.

Indicators	All hospitals	Hospital L	Hospital K
**Dispensing workload per month**			
No. pharmacists, mean (SD)	394 (17)	242 (10)	152 (10)
No. prescriptions, mean (SD)	463,587 (32,898)	283,873 (20,893)	179,714 (12664)
No. pharmacist-day, mean (SD)	9,016 (796)	5,546 (470)	3,470 (354)
Pharmacist dispensing workload (prescriptions per pharmacist working day), mean (SD)	52 (3)	51 (2)	52 (4)
**Prescription suggestions per month**			
No. prescription suggestions, mean (SD)	1,410 (324)	799 (208)	611 (138)
No. suggestions accepted by doctors, mean (SD)	1,252 (266)	720 (181)	532 (111)
No. CPOE-CSS-related errors, mean (SD)	499 (104)	287 (87)	212 (53)
No. CPOE-CSS -alerted suggestions, mean (SD)	389 (96)	250 (75)	139 (35)
No. Non-CPOE-CSS-alerted suggestions, mean (SD)	363 (110)	183 (55)	180 (66)
No. suggestions not accepted by doctors, mean (SD)	158 (84)	79 (42)	79 (42)
Prescription suggestion rate (suggestions per 10,000 prescriptions), mean (SD)	30 (7)	28 (7)	34 (8)
**Dispensing errors per month**			
No. dispensing errors, mean (SD)	388 (112)	366 (103)	22 (18)
No. wrong drug amount, mean (SD)	191 (50)	175 (45)	16 (15)
No. wrong drug, mean (SD)	197 (76)	191 (74)	6 (6)
Dispensing error rate (dispensing errors per 10,000 prescriptions), mean (SD)	8 (2)	13 (3)	1 (1)

CPOE-CSS, computerized physician order entry with clinical supportive systems; SD, standard deviations

There were negative associations between PDW and all PSR (adjusted rate ratio, aRR: 0.9786, 95%CI: 0.9744–0.9829), regardless of doctors’ acceptance (accepted suggestions: 0.9842, 95%CI: 0.9797–0.9888; non-accepted suggestions: 0.9357, 95%CI: 0.9228–0.9486), and types of CPOE-CSS assistance (alerted by CPOE-CSS: 0.9565, 95% CI: 0.9486–0.9644; not alerted by CPOE-CSS: 0.9707, 95% CI: 0.9621–0.9794). However, there was a positive correlation between PDW and PSR when pharmacists corrected entry errors to CPOE-CSS (1.0194, 95% CI: 1.0122–1.0266). There was also a negative correlation between PDW and all DER (0.9567, 95% CI: 0.9477–0.9658), regardless of types of dispensing errors (wrong drug dispensed, 0.9539, 95% CI: 0.9407–0.9672; wrong amount dispensed, 0.9547, 95% CI: 0.9426–0.9672) (**Table 2**).

**Table 2:** Multivariate Poisson regression model with generalized estimation equation for the association between dispensing workload and indicators of pharmacist performance.

Variables	aRR	(95% CI)		SE	P value
**1. Prescription suggestions**					
PDW	0.9786	0.9744	0.9829	0.0022	<0.0001
Hospital	1.2368	1.2218	1.2521	0.0063	<0.0001
Time	0.9851	0.9805	0.9893	0.0021	<0.0001
PDW*time	1.0004	1.0003	1.0005	0.0001	<0.0001
1-1. Suggestions accepted by doctors					
PDW	0.9842	0.9797	0.9888	0.0023	<0.0001
Hospital	1.1861	1.1707	1.2017	0.0067	<0.0001
Time	0.9926	0.9884	0.9970	0.0022	0.0010
PDW*time	1.0002	1.0002	1.0003	0.0001	<0.0001
1–1.1 Entry errors to CPOE-CSS					
PDW	1.0194	1.0122	1.0266	0.0036	<0.0001
Hospital	1.1452	1.1216	1.1692	0.0106	<0.0001
Time	1.0280	1.0211	1.0349	0.0034	<0.0001
PDW*time	0.9996	0.9995	0.9997	0.0001	<0.0001
1–1.2 CPOE-CSS-alerted suggestions					
PDW	0.9565	0.9486	0.9644	0.0042	<0.0001
Hospital	0.9179	0.8959	0.9404	0.0123	<0.0001
Time	0.9680	0.9600	0.9760	0.0042	<0.0001
PDW *time	1.0007	1.0005	1.0008	0.0001	<0.0001
1–1.3 Non-CPOE-CSS-alerted suggestions					
PDW	0.9707	0.9621	0.9794	0.0045	<0.0001
Hospital	1.6174	1.5793	1.6563	0.0122	<0.0001
Time	0.9719	0.9641	0.9798	0.0041	<0.0001
PDW*time	1.0007	1.0006	1.0009	0.0001	<0.0001
1–2. Suggestions not accepted by doctors					
PDW	0.9357	0.9228	0.9486	0.0070	<0.0001
Hospital	1.7057	1.6454	1.7683	0.0184	<0.0001
Time	0.9291	0.9175	0.9410	0.0064	<0.0001
PDW*time	1.0016	1.0013	1.0018	0.0001	<0.0001
**2. Dispensing errors**					
PDW	0.9567	0.9477	0.9658	0.0048	<0.0001
Hospital	0.0990	0.0943	0.1039	0.0246	<0.0001
Time	0.9716	0.9614	0.9819	0.0054	<0.0001
PDW*time	1.0005	1.0003	1.0007	0.0001	<0.0001
2–1. Wrong amount dispensed					
PDW	0.9539	0.9407	0.9672	0.0071	<0.0001
Hospital	0.0491	0.0447	0.0538	0.0470	<0.0001
Time	0.9778	0.9630	0.9928	0.0078	0.0038
PDW*time	0.9539	0.9407	0.9672	0.0002	0.0015
2–2. Wrong drug dispensed					
PDW	0.9547	0.9426	0.9669	0.0065	<0.0001
Hospital	0.1545	0.1459	0.1637	0.0293	<0.0001
Time	0.9616	0.9475	0.9758	0.0075	<0.0001
PDW*time	1.0007	1.0004	1.0010	0.0065	<0.0001

Abbreviation: aRR, adjusted rate ratio; CPOE-CSS, computerized physician order entry with clinical supportive systems; PDW, pharmacists’ dispensing workload

A significant interaction between PDW and time suggested that time modified the effect of PDW on the performance of pharmacy services, including PSR (p<0.0001) and DER (p<0.0001). The impact of PDW on all PSR was nearly the same in different time periods; however, there were negative associations between PDW and all DER during 2012–2015 (0.9674, 95%CI: 0.9613–0.9738), while positive associations were found during 2016–2018 (1.0086, 95%CI: 1.0003–1.0169) (**Table 3**).

**Table 3:** Association between dispensing workload and indicators of pharmacist performance, stratified by time period*.

	Adjusted RR* (95% CI)
**1. Prescription suggestions**	
Time period 1	0.9674 (0.9641–0.9708)
Time period 2	0.9784 (0.9752–0.9818)
1–1. Suggestions accepted by doctors	
Time period 1	0.9728 (0.9692–0.9765)
Time period 2	0.9806 (0.9771–0.9818)
1–1.1 Entry errors to CPOE-CSS	
Time period 1	0.9802 (0.9743–0.9861)
Time period 2	0.9648 (0.9593–0.9703)
1–1.2 CPOE-CSS-alerted suggestions	
Time period 1	0.9567 (0.9506–0.9629)
Time period 2	0.9860 (0.9792–0.9927)
1–1.3 Non-CPOE-CSS-alerted suggestions	
Time period 1	0.9823 (0.9753–0.9897)
Time period 2	0.9947 (0.9888–1.0008)
1–2. Suggestions not accepted by doctors	
Time period 1	0.9287 (0.9191–0.9384)
Time period 2	0.9614 (0.9516–0.9713)
**2. Dispensing errors**	
Time period 1	0.9674 (0.9613–0.9738)
Time period 2	1.0086 (1.0003–1.0169)
2–1. Wrong amount dispensed	
Time period 1	0.9783 (0.9698–0.9869)
Time period 2	1.0009 (0.9888–1.0133)
2–2. Wrong drug dispensed	
Time period 1	0.9549 (0.9457–0.9641)
Time period 2	1.0151 (1.0038–1.0266)

Abbreviation: RR, rate ratio; CPOE-CSS, computerized physician order entry with clinical supportive systems; PDW, pharmacists’ dispensing workload

*We defined two different time periods as 2012–2015 and 2016–2018, and the estimates were adjusted by individual hospital.

## Discussion

A maximum of 40 prescriptions per day per pharmacist is stipulated by law in Japan[22], which is a lower volume than that handled by pharmacists in medical centers in Taiwan (40 inpatient prescriptions or 70 outpatient prescriptions per pharmacist per day). We found the dispensing volume of pharmacists in Taiwan to be higher than suggested (52 ± 3 prescriptions per pharmacist working days), highlighting the need to evaluate the clinical services of pharmacists under such a high dispensing burden. As a result of efforts by government and hospital administrators in Taiwan to maintain an optimal pharmacy workload, we found that the PDW decreased during the 2012–2018 study period in these two large medical centers. Although we did find that there was an increase in prescription volume during this period, the monthly number of pharmacists in these hospitals also increased by 13% (**S2 Fig**) in order to provide better pharmaceutical care.

There seems to be an increasing trend in PSR during the study period. Like in other developed countries with aging populations[23], the number of hospital outpatient visits has been increasing every year and has created a higher demand for pharmacy services in Taiwan. One report indicated that the average number of hospital visits per person per year was 13.9 in 2007 in Taiwan, which was higher than in most other countries[24]. Previous studies have also reported that a high percentage of Taiwanese people have had the experience of visiting different healthcare facilities on the same day, which may increase the risk of developing adverse drug reactions and incurring duplicate and unnecessary medications, polypharmacy, and drug-drug interactions[25–27]. With the introduction and continuous improvement of CPOE-CSS in the study hospitals, pharmacists are better able to identify potential drug-related problems. Moreover, the pharmacy profession in Taiwan has developed patient-oriented courses which help to improve the clinical competency of pharmacists[28], such as by working with other health professionals. Based on our results, we highlight the irreplaceable and expanding role of pharmacists in maintaining the quality of medication use.[29,30]

Consistent with previous studies from the US and the UK[16,17], we found that higher dispensing workloads were associated with lower rates of prescription suggestions. We found the aRR was about 0.98 between PDW and PSR based on the regression model, implying that an increase in PDW by one prescription per pharmacist per day would reduce the PSR by 2%. We found the PDW decreased from 53 to 47 from 2012–2018, while the PSR increased 12% within the study period **(Fig 2)**. These findings encourage the efforts to reduce PDW to free up time for pharmacists to review prescriptions. Although previous studies have shown the implementation of CPOE-CSS to be associated with a decline of more than 50% in preventable adverse drug events in hospital-related settings[31], we found that when they were busy with high prescription volumes, pharmacists may fail to review drug-related problems, including drug allergies or drug-drug interactions, even when automatically alerted by CPOE-CSS. While it is widely believed that health information technology can enhance medication safety and improve quality of care, Korb-Savoldelli V et al reported that the CPOE-CSS contributed to 6–78% of medication errors made by physicians during prescribing[32]. Since entry errors to CPOE-CSS are easier to identify, we found that pharmacists could still provide prescription suggestions under a high dispensing volume.

Dispensing errors have long been among the biggest challenges for pharmacists, especially in Taiwan due to the large numbers of prescriptions and lack of assistance from pharmacy technicians. There are many factors involved in a dispensing error, but James KL et al found heavy workload to be the most important factor contributing to dispensing errors[15]. To maintain the accuracy of dispensing, pharmacy administrators have developed strategies to minimize possible dispensing errors. For example, pharmacists have used reminders for high error-rate drugs with similar names, packages and dosages to prevent look-alike dispensing errors[33]; pharmacists have also attended routine educational meetings to review high frequency dispensing errors and their possible causes and solutions. Moreover, pharmacists have used computer systems to improve dispensing efficiency to make available more time for prescription validations[34]. Automatic dispensing machines are currently able to deal with more than one hundred common drugs. Given the many interventions to reduce the impact of high prescription volumes on dispensing errors, we report that there appears to be a decreasing trend of dispensing errors in this study.

We found the DER overall slightly increased during 2012–2018 **(Fig 2),** but the associations between PDW and DER differed in the time periods of 2012–2015 (aRR: 0.96) and 2016–2018 (aRR: 1.01). The implication that increasing PDW by one prescription per pharmacist per day would reduce DER by 4% is deceptive, and more likely reflects that higher PDW may have led to lower reports of dispensing errors in previous years. One of the most likely explanations for the discrepancy between the different time periods is that access to the dispensing error reporting system may not have been convenient in the previous years, compared to the later years, so the DER may have been lower, especially while pharmacists were busy. In addition, by 2016, study hospitals had strengthened the medical informatics system and completed full clinical decision support systems accredited following the sixth-stage HIMSS Electronic Medical Record Adoption Model (EMRAM). The improvement of computer systems probably rendered the dispensing error reporting system more accessible. As a result, we found increasing PWD by one prescription per pharmacist per day increased DER by about 1% during 2016–2018. The effects were lower than we expected, because dispensing errors could also result from underlying causes other than prescription volumes, e.g., the quality of dispensing environment, personal skills and pharmaceutical knowledge[35]. Our findings may reflect the trade-off between dispensing medications and review of prescriptions, whereby pharmacists may focus their efforts on maintaining the quality of dispensing while under a high workload situation.

Pharmacists play multiple important roles in reviewing prescriptions, dispensing, education of patients and healthcare providers, and in drug management. The findings provide important background leverage to our efforts to reduce dispensing workload of pharmacists, to provide opportunities and time for pharmacists to complete other important tasks. In addition to the rational use of machinery and computers to facilitate pharmacy services, we encourage the routine evaluation of pharmacists’ workload and the recruitment of sufficient numbers of pharmacists to maintain medication quality and safety.

In this study, we analyzed spontaneous reports from 2012 to 2018 from the two largest medical centers in Taiwan to report on the impact of pharmacists’ workload on pharmacy services. However, similar to other spontaneous reporting databases, potential threats to our study findings included the data quality such as under-reporting bias and internal validity[36]. To increase the reporting rates, we had encouraged each report by a reward of up to 100 NT dollars and work credits. Moreover, we made efforts to educate pharmacists and to emphasize the importance of this reporting system and also made upgrades to systems and facilities. However, we could not assess the levels of under-reporting of prescription suggestions and dispensing errors from pharmacists, but we consider this bias to be minor in our observation periods based on our findings that trends of PSR and DER were consistent over time. Also, we only used the review of prescriptions and correct dispensing as indicators for pharmacist services, which may not cover other administrative and pharmaceutical services of pharmacists, such as patient education. Furthermore, our findings from the two largest medical centers in Taiwan may not be generalizable to other hospitals. Finally, like all analyses using data from spontaneous reporting systems, we may not have precisely estimated the incident PSR and DER [37].

## Conclusion

Our findings indicate that reduced dispensing workload could increase pharmacists’ capacity to review prescriptions and also decrease dispensing errors, especially in 2016–2018. This study provides a foundation for the determination of pharmacy workload to improve the quality of healthcare.

## Supporting information

S1 Fig The trends of PDW, PSR and DER between 2012 and 2018 in individual hospitals. (TIFF)

S2 Fig The trends of monthly prescription volume and the number of pharmacist between 2012 and 2018. (TIFF)

S1 Table Original data of PDW, PSR and DER in study hospitals during the study period. (DOCX)
