# Supplementary figures and images for: Workload of pharmacists and the performance of pharmacy services
Source: PLoS One. 2020 Apr 21;15(4):e0231482. doi: 10.1371/journal.pone.0231482 (PMC7173874; doi:10.1371/journal.pone.0231482)

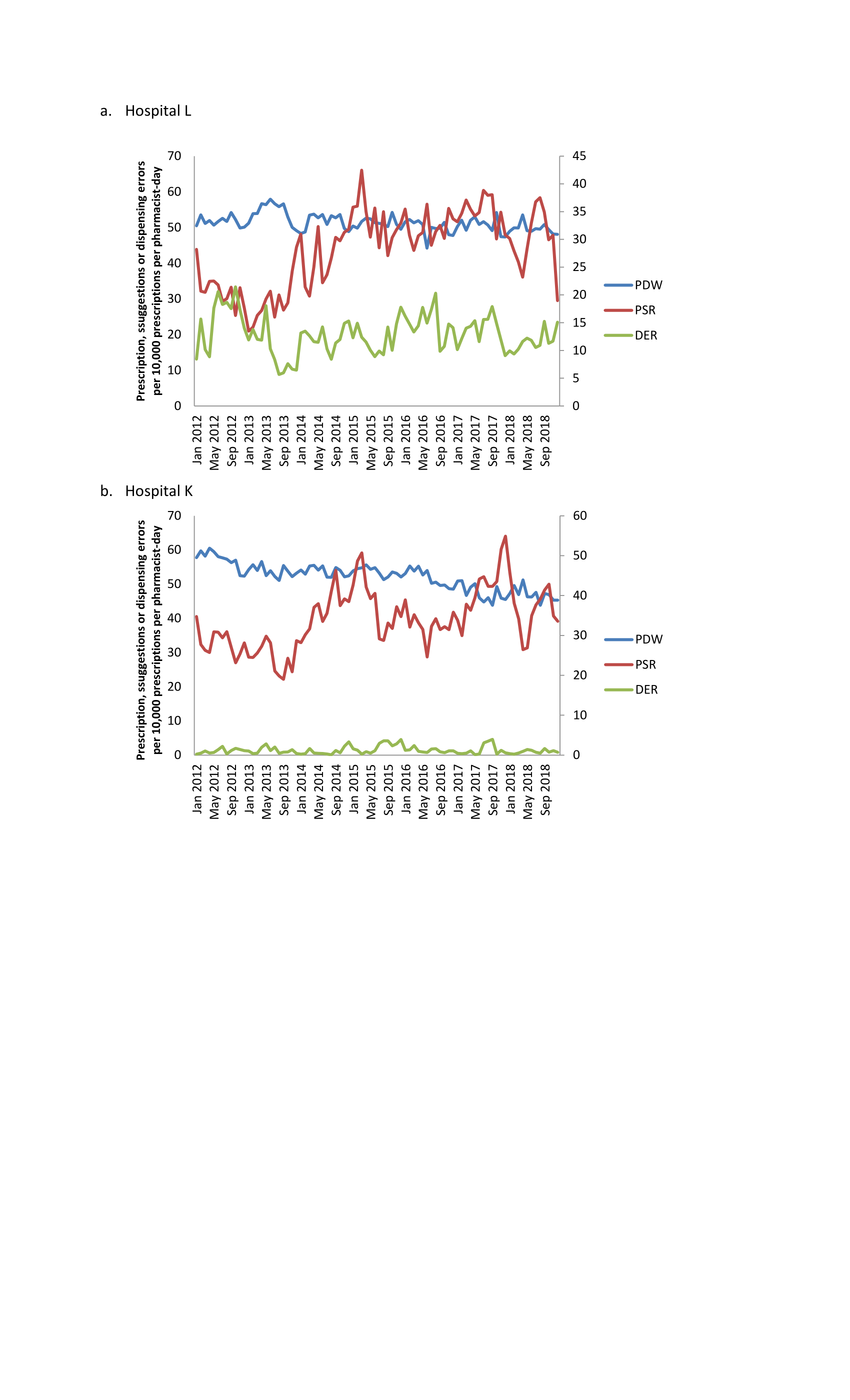

Supplement: S1 Fig — (TIFF) [file pone.0231482.s001.tiff]

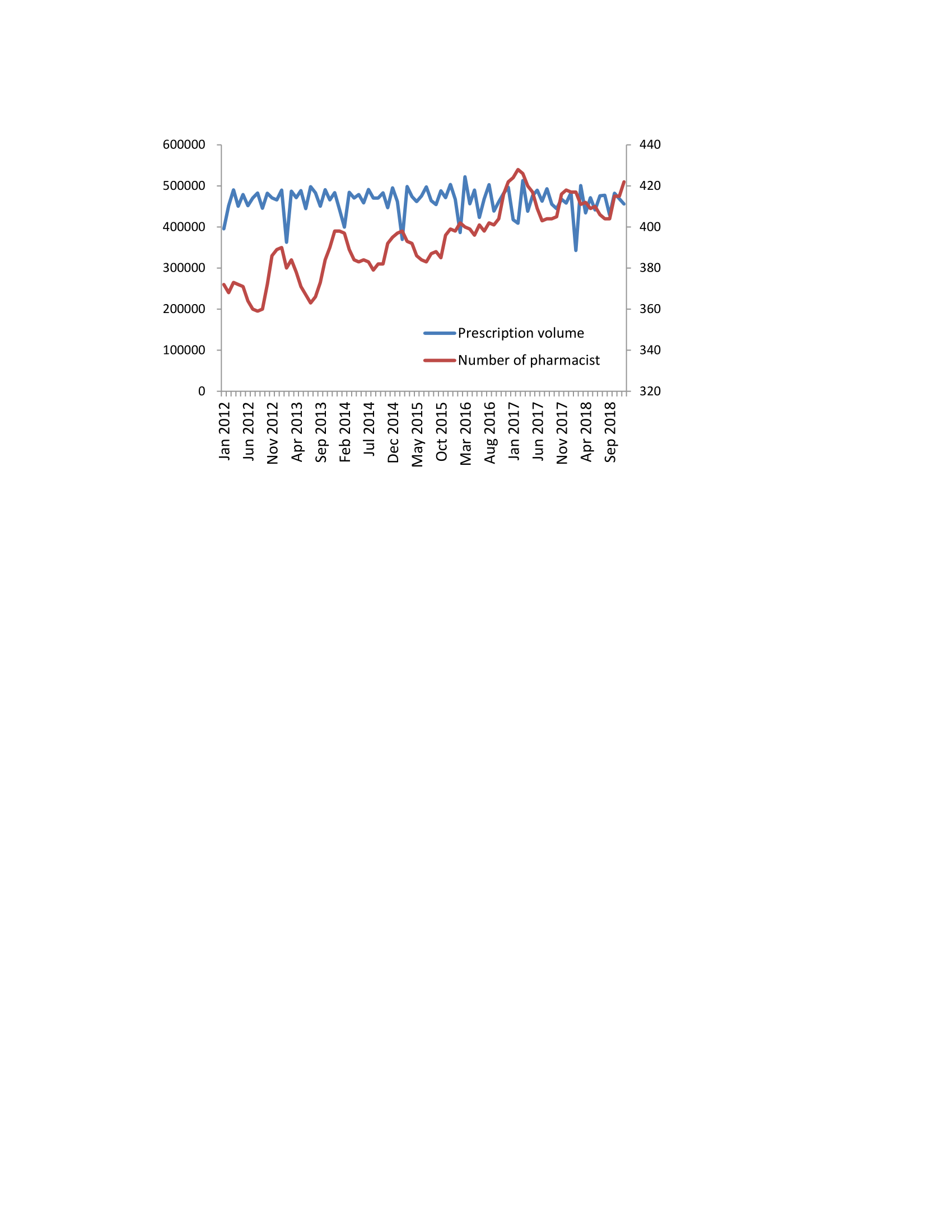

Supplement: S2 Fig — (TIFF) [file pone.0231482.s002.tiff]
