# Supplementary material for: Workload of pharmacists and the performance of pharmacy services
Source: PLoS One. 2020 Apr 21;15(4):e0231482. doi: 10.1371/journal.pone.0231482 (PMC7173874; doi:10.1371/journal.pone.0231482)
Supplement: S1 Table — (DOCX) [file pone.0231482.s003.docx]

**S1 Table. Original data of PDW, PSR and DER in study hospitals during the study period.**

| Month | PDW | PSR | DER |
| --- | --- | --- | --- |
| Jan 2012 | 53.1 | 30.8 | 5.2 |
| Feb 2012 | 55.8 | 23.4 | 9.7 |
| Mar 2012 | 53.6 | 22.7 | 6.6 |
| Apr 2012 | 55.0 | 23.7 | 5.6 |
| May 2012 | 53.8 | 25.8 | 11.0 |
| Jun 2012 | 54.0 | 25.3 | 13.2 |
| Jul 2012 | 54.5 | 22.9 | 12.0 |
| Aug 2012 | 53.7 | 23.8 | 11.5 |
| Sep 2012 | 55.0 | 23.5 | 11.3 |
| Oct 2012 | 54.0 | 19.0 | 13.7 |
| Nov 2012 | 50.8 | 22.9 | 11.1 |
| Dec 2012 | 50.9 | 21.7 | 9.0 |
| Jan 2013 | 52.4 | 17.8 | 7.7 |
| Feb 2013 | 54.6 | 18.2 | 8.5 |
| Mar 2013 | 54.0 | 19.9 | 7.6 |
| Apr 2013 | 56.7 | 21.1 | 8.0 |
| May 2013 | 54.8 | 23.3 | 12.2 |
| Jun 2013 | 56.4 | 23.5 | 6.8 |
| Jul 2013 | 54.9 | 18.0 | 5.9 |
| Aug 2013 | 53.9 | 19.9 | 3.7 |
| Sep 2013 | 56.2 | 17.9 | 3.9 |
| Oct 2013 | 53.3 | 20.8 | 5.0 |
| Nov 2013 | 50.8 | 22.9 | 4.6 |
| Dec 2013 | 50.6 | 28.6 | 4.1 |
| Jan 2014 | 50.5 | 29.8 | 8.0 |
| Feb 2014 | 50.3 | 24.9 | 8.3 |
| Mar 2014 | 54.2 | 24.4 | 8.4 |
| Apr 2014 | 54.4 | 29.7 | 7.2 |
| May 2014 | 53.2 | 34.5 | 7.2 |
| Jun 2014 | 54.3 | 26.6 | 8.8 |
| Jul 2014 | 51.3 | 28.3 | 6.4 |
| Aug 2014 | 52.8 | 32.1 | 5.2 |
| Sep 2014 | 53.5 | 36.6 | 7.3 |
| Oct 2014 | 53.8 | 32.7 | 7.5 |
| Nov 2014 | 50.6 | 34.3 | 10.0 |
| Dec 2014 | 50.2 | 34.4 | 10.6 |
| Jan 2015 | 51.7 | 38.4 | 8.1 |
| Feb 2015 | 51.6 | 41.0 | 9.5 |
| Mar 2015 | 52.8 | 45.7 | 7.6 |
| Apr 2015 | 53.8 | 37.8 | 7.4 |
| May 2015 | 53.1 | 33.8 | 6.4 |
| Jun 2015 | 52.7 | 37.6 | 5.8 |
| Jul 2015 | 51.9 | 28.7 | 7.2 |
| Aug 2015 | 51.0 | 32.6 | 7.0 |
| Sep 2015 | 50.9 | 29.5 | 10.0 |
| Oct 2015 | 54.0 | 30.9 | 7.0 |
| Nov 2015 | 51.7 | 33.9 | 10.1 |
| Dec 2015 | 50.5 | 33.6 | 12.4 |
| Jan 2016 | 52.2 | 36.8 | 10.4 |
| Feb 2016 | 53.4 | 31.2 | 9.5 |
| Mar 2016 | 52.2 | 30.8 | 9.1 |
| Apr 2016 | 53.2 | 31.6 | 9.2 |
| May 2016 | 51.5 | 31.3 | 11.2 |
| Jun 2016 | 48.0 | 31.3 | 8.7 |
| Jul 2016 | 50.1 | 30.2 | 11.2 |
| Aug 2016 | 50.0 | 32.5 | 13.0 |
| Sep 2016 | 49.7 | 32.1 | 6.3 |
| Oct 2016 | 50.8 | 30.9 | 6.8 |
| Nov 2016 | 48.3 | 34.0 | 9.4 |
| Dec 2016 | 48.1 | 34.5 | 9.1 |
| Jan 2017 | 50.5 | 33.4 | 6.3 |
| Feb 2017 | 51.7 | 32.8 | 7.6 |
| Mar 2017 | 48.2 | 37.4 | 8.8 |
| Apr 2017 | 50.9 | 35.8 | 9.2 |
| May 2017 | 51.8 | 36.2 | 9.4 |
| Jun 2017 | 48.9 | 38.4 | 7.2 |
| Jul 2017 | 48.8 | 41.0 | 10.9 |
| Aug 2017 | 48.8 | 39.6 | 11.0 |
| Sep 2017 | 47.0 | 39.7 | 12.7 |
| Oct 2017 | 52.2 | 35.2 | 9.3 |
| Nov 2017 | 46.9 | 41.2 | 7.9 |
| Dec 2017 | 46.6 | 39.9 | 5.9 |
| Jan 2018 | 48.3 | 36.1 | 6.3 |
| Feb 2018 | 49.8 | 31.8 | 5.8 |
| Mar 2018 | 48.7 | 29.0 | 6.6 |
| Apr 2018 | 52.7 | 24.4 | 7.5 |
| May 2018 | 48.0 | 27.7 | 8.1 |
| Jun 2018 | 47.8 | 33.7 | 7.8 |
| Jul 2018 | 48.8 | 37.1 | 6.7 |
| Aug 2018 | 47.2 | 38.1 | 7.0 |
| Sep 2018 | 49.4 | 37.4 | 10.0 |
| Oct 2018 | 48.4 | 34.9 | 7.3 |
| Nov 2018 | 47.0 | 32.3 | 7.7 |
| Dec 2018 | 47.0 | 24.6 | 9.6 |
